# Dementia inclusive retail outlets: a scoping review of strategies to support people living with dementia in the retail industry

**DOI:** 10.3389/frdem.2026.1852323

**Published:** 2026-08-17

**Authors:** Megan Rose Readman, Sarah Fox, Abigail Godfrey, Annabel Farnood, Sarah K. Smith, Olivia Luijnenburg, Lettie Wareing, Manjot Brar, Rebecca Ramos, Megan Polden

**Affiliations:** 1 National Institute of Health and Social Care Research Applied Research Collaboration North West Coast, Liverpool, United Kingdom; 2 Department of Psychology, Lancaster University, Lancaster, United Kingdom; 3 Department of Primary Care and Mental Health, University of Liverpool, Liverpool, United Kingdom; 4 National Institute of Health and Social Care Research Applied Research Collaboration Greater Manchester, Manchester, United Kingdom; 5 Cicely Saunders Institute of Palliative Care Policy & Rehabilitation, London, United Kingdom; 6 National Institute for Health and Care Research Applied Research Collaboration South London, London, United Kingdom; 7 National Institute of Health and Care Research Health and Social Care Workforce Research Unit, The Policy Institute, King’s College London, London, United Kingdom; 8 Department of Health Research, Lancaster University, Lancaster, United Kingdom

**Keywords:** community participation, dementia, dementia-friendly communities, retail, shopping

## Abstract

**Background:**

Supermarket shopping is a valued everyday activity that supports autonomy and social engagement for people living with dementia (PLWD). However, engagement with retail environments can be challenging due to difficulties with product recognition and selection, navigation, and sensory overwhelm. Despite increasing efforts to develop dementia-friendly communities, there is currently no comprehensive synthesis of evidence examining how PLWD experience retail environments or which strategies effectively support their engagement.

**Methods:**

PubMed, PsycINFO, CINAHL, MEDLINE, and EMBASE were searched (2007–2025) for quantitative, qualitative and mixed-methods studies examining (1) retail engagement experiences of PLWD and/or informal carers, (2) practical recommendations for the retail sector, and (3) initiatives currently implemented in shops. Grey literature searches using Google supplemented these sources. Data were synthesised using reflexive thematic analysis.

**Results:**

Ten academic and 59 non-academic resources, including guidance and reports of initiatives, were identified. Relating to the experience of retail engagement for PLWD four overarching themes were identified: (1) Reliance on Familiarity and Predictability, (2) Cognitive Demands Before and During Shopping, (3) Sensory Overload and Time Pressure, (4) Payment, Technology, and Feelings of Exposure. Practical recommendations consistently highlight the importance of both person-centred approaches and environmental adaptations, which are reflected in many initiatives currently implemented in shops.

**Conclusion:**

Despite the centrality of retail environments to community engagement for PLWD cognitive, sensory and social barriers impede independent positive engagement with these environments. Although dementia-friendly initiatives have been widely implemented across the retail sector, there remains a critical gap in rigorous evaluation of their effectiveness and long-term impact. Furthermore, some interventions may inadvertently contribute to the stigmatisation of PLWD, highlighting the need for inclusive, stigma-reducing strategies that support autonomy and participation.

**Registration:**

This review was pre-registered on the Open Science Framework (https://osf.io/gfyva/).

## Background

Dementia is a progressive neurological condition characterised by a decline in cognitive functions, such as memory, thinking, and language, alongside declining capability to perform everyday activities, severe enough to interfere with an individual’s independence and quality of life (Livingston et al., 2024). Dementia is a growing public health concern, with over 55 million people currently living with the condition globally (World Health Organization, 2023) with this number projected to increase to 139 million by 2050 (Alzheimer’s Disease International, 2020). Despite significant scientific advancements, including the emergence of potentially disease-modifying therapies such as Donanemab and Lecanemab, dementia remains a progressive and currently incurable condition (Livingston et al., 2024). Consequently, supporting people living with dementia (PLwD) to live well is a central priority worldwide.

Home-based care, wherein the PLWD remains living at home, often with family and friends acting as unpaid carers, alongside professional domiciliary care where required, is widely regarded as the preferred model of support for PLWD (Leverton and Pui Kin Kor, 2023). This approach supports the maintenance of familiar routines, familiar places, social relationships, and a sense of personal identity, all of which are closely associated with wellbeing and quality of life in dementia (O’Rourke et al., 2015). However, as the condition progresses, cognitive and functional decline typically results in increasing support needs. These commonly include assistance with basic activities of daily living (e.g., bathing, dressing, and eating), instrumental activities of daily living (e.g., financial management, shopping, and meal preparation), and support to engage in valued social and leisure activities (Giebel et al., 2014, 2015). As support needs increase, individuals often experience a gradual reduction in engagement with activities outside the home (Clark and Phillipson, 2021). Consequently, although many people with dementia continue to reside within their local communities, they may experience a progressive contraction of their accessible lived environments, often described as a “shrinking world” (Duggan et al., 2008).

Importantly, prior evidence suggests that reductions in spatial and social engagement do not have to be an inevitable consequence of dementia. Rather, creating well-designed and supportive communities through altering modifiable environmental and social factors, including neighbourhood design, the availability of supportive infrastructure, and the presence of inclusive community practises and initiatives (Ward et al., 2022) may mitigate the functional and social impacts of dementia, enabling continued autonomy, social connectedness, and meaningful participation in everyday life.

Dementia-friendly communities, defined as “places or cultures in which people living with dementia (PLWD) and their carers are empowered, supported, and included in society, with their rights recognised and their potential valued” (Alzheimer’s Disease International, 2016), constitute a structured and intentional response to this challenge. Central to these initiatives is the recognition of PLWD and their carers as valued members of their local communities. This is achieved through coordinated efforts to raise public awareness, reduce stigma, and promote inclusion, alongside environmental adaptations, service-level changes, and workforce training designed to ensure that community spaces, services, and social interactions are accessible, understandable, and supportive (Hebert and Scales, 2019; Hung et al., 2021). One clear example of national action in support of dementia-friendly communities is the United Kingdom’s Prime Minister’s Challenge on Dementia 2020 (UK Government, 2015). Delivered in partnership with the Alzheimer’s Society, this policy included a commitment to encourage and support businesses to become dementia friendly through the promotion of dementia awareness training via programmes such as Dementia Friends, as well as the development of sector-specific dementia-friendly business charters. While there remains a paucity of evaluations examining the impact of dementia-friendly training within business settings, existing evidence suggests that Dementia Friends training can increase knowledge and confidence among undergraduate nursing students (Mitchell et al., 2017). Furthermore, this training was perceived positively among school-aged children in the UK (Farina et al., 2020).

Retail environments, such as supermarkets and high streets, are particularly important community spaces. Shopping is a frequent and highly valued activity that enables people living with dementia (PLWD) to maintain autonomy, connection, control, and social engagement. Indeed, qualitative studies suggest that shopping trips function not only as instrumental tasks but also as important opportunities for social connection, sensory engagement, and continuity of identity for people living with dementia (Brorsson et al., 2011, 2013; Clark and Phillipson, 2021; Fox et al., 2025a). However, instrumental activities of daily living, such as supermarket shopping, are often impaired in PLWD as cognitive symptoms progress, increasing the complexity and reducing the accessibility of these activities (Giebel et al., 2014). Prior literature suggests that numerous elements, including remembering to bring the necessary items to the shop, changes in the location of products, organisational layout (including physical and sensory aspects), product selection, and payment processes, can cause substantial challenges for PLWD (Brorsson et al., 2011, 2013; Johansson et al., 2011). Therefore, it is important that people working in the retail sector are aware of the challenges PLWD face and how they can support them to continue with this activity for as long as possible.

Despite the growing emphasis on dementia-friendly initiatives, there is no comprehensive synthesis of the evidence regarding how PLWD experience retail environments, or which strategies effectively support their engagement. To this end, this scoping review aims to synthesise evidence regarding the experiences of PLWD when engaging with the retail industry and strategies that have been employed to support PLWD to access and utilise retail environments. This review will provide insight into the current evidence base, identify gaps for future research, and inform both policy and practise in creating inclusive, dementia-friendly retail spaces.

## Methods

This scoping review was pre-registered, on the Open Science Framework prior to completion (Fox et al., 2025b). The OSF pre-registration contains documentation of the search strategy and planned synthesis. Implementation adhered to the pre-registered protocol and is summarised below.

### Search strategy

Given the nature of the research question, many relevant materials are likely to be produced by charities and organisations rather than being published in academic journals, and so would not be captured by traditional database searching. Consequently, we employed a comprehensive search strategy, encompassing both multidisciplinary academic databases and grey literature searches using Google to capture reports, guidelines, and resources not indexed in traditional databases.

#### Academic database searching

To address the multidisciplinary nature of the research question, a comprehensive literature search using PubMed, PsychInfo, CINHAL, Medline, and EMBASE, was conducted on 27.02.2025. EMBASE and PsychInfo were selected as they include pre-print databases such as BioArXiv and MedArXiv, and theses, thereby ensuring grey literature (theses, conference abstracts etc.) was captured. The Alzheimer’s Society began the UK’s first Dementia Friendly Communities programme and launched the national Dementia Friends programme in 2012 (UK Government, 2013). Therefore, to ensure any literature that contributed to the initiation of these programmes and any papers that followed are captured database searches were restricted to 2007–2025.

For each database, three independent search strings, one for the population of interest (i.e., dementia), one pertaining to the phenomenon of interest (i.e., retail), and one pertaining to the more general concept of inclusive communities was applied. These independent search strings were combined using Boolean operators (“AND” and “OR”). Each search string comprised dictionary terms specific to the database in question and free-text search terms. Therefore, the search strings applied to each database differed only in terms of the database specific dictionary terms applied. Where dictionary terms are applied the decision to explode these terms was made on a case-by-case basis by considering the relevance of the exploded terms to the review question (see Supplementary Table S1 for applied search strings).

The sensitivity of the search strategy was assessed using three key papers that were *a priori* deemed to be particularly relevant to our research question (Brorsson et al., 2011, 2013, 2020). The initial search string proved to be sensitive for all databases and hence was not updated further. In circumstances in which we could not access the relevant record, The University of Liverpool Library requested access.

#### Grey literature searching

To increase the comprehensiveness of the search strategy a comprehensive grey literature search using Google was conducted on 28.03.2025. As these conventional search engines do not allow for multiple searches to be combined, nine individual searches, each using one string, were conducted (see Supplementary Table S2 for applied search strings).

### Inclusion criteria

As this review aimed to synthesise evidence regarding the experiences of PLWD when engaging with the retail industry and strategies that have been employed to support PLWD to access and utilise retail environments, a sample, phenomenon of interest, design, evaluation, research type (SPIDER) eligibility criteria was applied. Eligible studies and/or guidance documents were those which (1) included a population of PLWD, and/or informal carers for someone living with dementia (sample); (2) focused on the experience of engaging with the retail industry for people living with or caring for someone living with dementia, or strategies (or interventions) to support PLWD to engage with the in store retail industry (phenomenon of interest); (3) employed any methodological design (design); evaluated PLWD and/or informal carers experiences or the outcomes of interventions (evaluation); and (4) were either quantitative (e.g., surveys), qualitative (e.g., focus groups, interviews), or mixed-methods (e.g., interviews and survey) studies (research type).

Screening was conducted in two phases: (1) title and abstract screening, and (2) full text screening. The inclusion criteria differed depending on the phase of screening (see Table 1 for screening criteria).

**Table 1:** Inclusion criteria applied at the level of title and abstract and full text screening for both academic and non-academic articles.

Screening	Criteria
Academic articles	
Title/Abstract	The abstract is written in the English Language. An original study (i.e., no review articles). The study involves a population of people living with dementia, and/or informal carers for someone living with dementia. There is mention of engaging with the retail industry for people living with or caring for someone living with dementia OR there is mention of strategies (or interventions) to support people living with dementia to engage with the retail industry.
Full text	Full text is written in the English language. People living with dementia, or people caring for people with dementia’s experiences of engaging within the retail industry is discussed OR strategies to overcome barriers to engaging with the retail industry are discussed.
Non-academic articles	
Title	The title is written in the English Language. The article refers to people living with dementia. There is mention of engaging with the retail industry for people living with dementia.
Full text	The article is written in the English language. The article refers specifically people living with dementia. The article refers to policies and/or guidance on how to support people living with dementia in the retail industry. Published within the past 10 years.

### Screening

All authors were involved in the screening process with each article being screened by two independent reviews in parallel, blinded to each other’s decisions. Any discrepancies that arose between the two reviewers were resolved by a third reviewer. The full text of all title/abstracts that were deemed to be potentially relevant were then screened by two independent reviewers, again blinded to each other’s decisions. Any discrepancies were resolved by a third reviewer. All academic screening was conducted and managed using CADIMA (Kohl et al., 2018) and all non-academic screening was conducted and managed using Microsoft Excel. Prior to the completion of any screening all articles were deduplicated. For academic articles this occurred using the automated function in CADIMA and checked manually by MRR, and for non-academic articles this was conducted using the automatic duplication feature embedded within excel and manually checked by MRR.

All studies meeting these inclusion criteria were checked for retraction using the retraction watch database^1^, with no papers having been retracted.

### Data extraction

Data pertaining to academic articles was extracted by MRR and checked by a second reviewer (AG). Data pertaining to non-academic materials was extracted by RR and checked by a second reviewer (MRR). If discrepancies arose, the inconsistency was resolved by a third reviewer (MP). For academic articles, the following data were extracted into an excel spreadsheet; study authors, year of study, sample size (*n*), population sampled [type of participant (PLWD/carers) and (*n*)], research method (quantitative, qualitative, mixed methods), summary of observations. For non-academic materials the following data was extracted; author(s) (this may be the charity or the policy administrator), year of policy or guidance, summary of findings/recommendations (see Supplementary Tables S3, S4 for data extraction).

### Assessment of methodological quality

The methodological quality of all academic articles that passed through full-text screening was assessed using the mixed methods appraisal tool (MMAT) (Hong et al., 2018). As there are no appropriate tools to assess the quality of non-academic policy and best practise guidance, we did not conduct a thorough assessment of the methodological quality of non-academic materials. To enhance transparency regarding publication quality, information relating to journal indexing/ranking was extracted using the Scimago Journal & Country Rank database (Scimago Journal & Country Rank, n.d.).

### Data synthesis

Data were synthesised based on whether they (1) relate to the experience of engaging with the retail industry for PLWD or (2) relate to support (interventions) that facilitate engagement with the retail industry for PLWD. Extracted data were analysed using the principles of thematic analysis (Clarke and Braun, 2017). Initial coding was conducted independently by two members of the research team (MR and AG) to enhance rigour and reduce bias. Codes were then compared and discussed. Related codes were subsequently collated into themes, which were reviewed and refined through discussion within the wider research team to ensure coherence.

## Results

**Figure 1:**
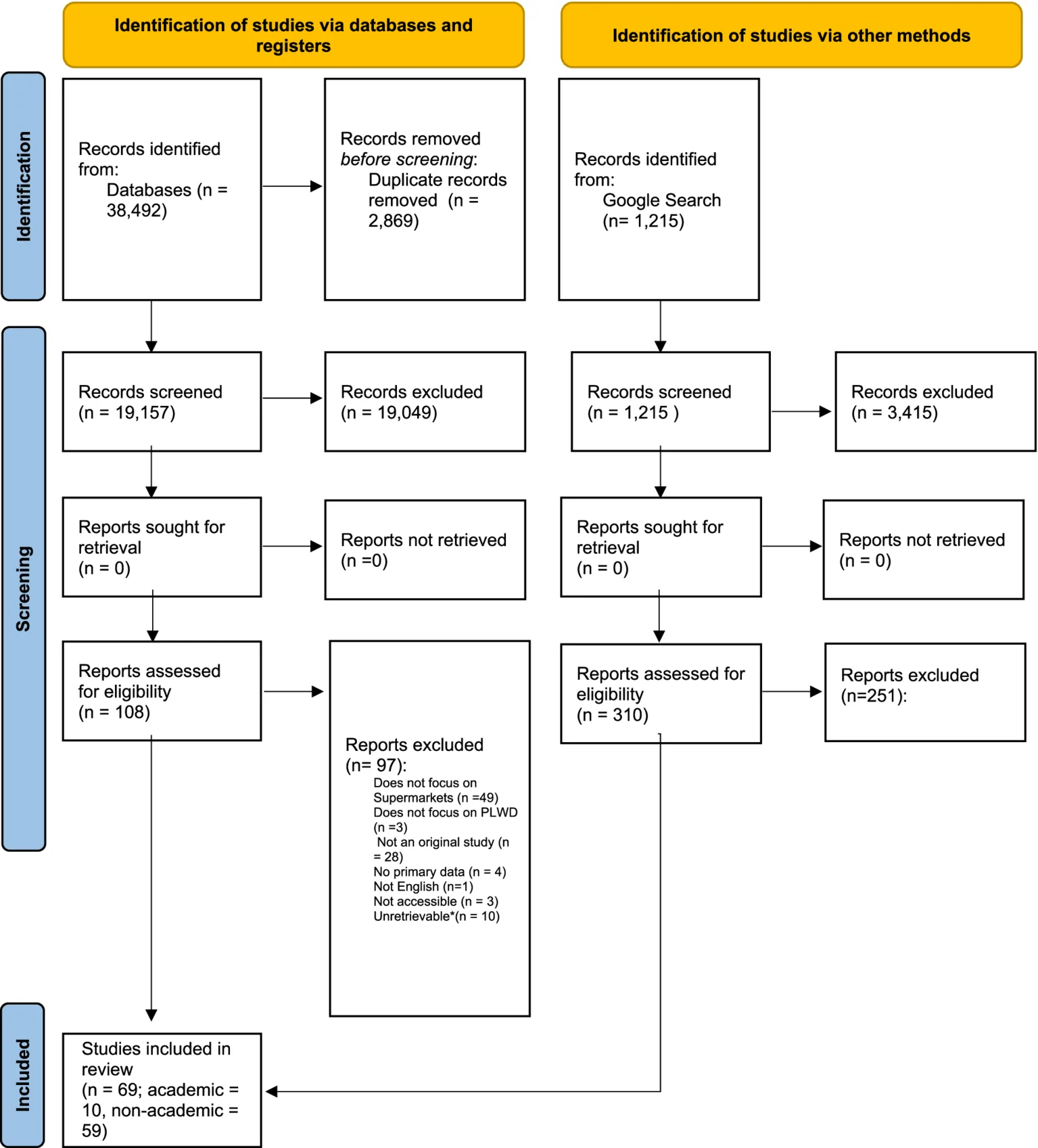
PRISMA flowchart outlining number of papers excluded at each stage of screening. Only the first reason for exclusion is reported. From: Page et al. (2021).

Of the 19,157 deduplicated academic records ten relevant academic studies, comprising 2,012 participants (PLWD *n* = 1,172; carers *n* = 734; caregiver and care receiver dyads *n* = 41; shop owners *n* = 12) (note the sample size was not reported in two studies) were identified (see Figure 1 for PRISMA diagram). All studies employed a qualitative methodology, including interviews (*n* = 8), photograph analysis (*n* = 2), focus groups (*n* = 1), content analysis (*n* = 1), open-ended survey responses (*n* = 1), and participant observations (*n* = 1), (note some studies employed more than one methodology hence why the total *n* by methodology does not equal the number of studies). Eight of the ten academic studies related to the lived experience of engaging with the retail industry for PLWD (Brorsson et al., 2011, 2013, 2020; Crampton and Eley, 2013; Silverman, 2021; Hebert, 2019; Wu et al., 2019; Quinn et al., 2022). In addition, nine studies made recommendations as to interventions to facilitate engagement with the retail industry for PLWD (Brorsson et al., 2013, 2020; Crampton and Eley, 2013; Silverman, 2021; Hebert, 2019; Wu et al., 2019; Ward et al., 2018; Igarashi et al., 2020; Quinn et al., 2022).

Of the 1,561 deduplicated non-academic materials 59 relevant non-academic materials were identified. Articles were published between 2012 and 2024, with a peak in articles being published in 2017 and 2018. All articles covered either (1) challenges PLWD faced in UK-based supermarkets (*n* = 2), (2) guide resources detailing practical recommendations for the retail sector, issued by retailers, charities or dementia organisations (*n* = 21), or (3) or initiatives in place in shops (*n* = 36).

### The experience of engaging with the retail industry for PLWD

Eight academic studies (Brorsson et al., 2011, 2013, 2020; Crampton and Eley, 2013; Silverman, 2021; Hebert, 2019; Wu et al., 2019; Quinn et al., 2022) and two non-academic outputs (Juste, 2023; Healthcare News, 2017) explored the retail experiences of PLWD. Four interrelated analytical themes were identified: (1) Reliance on Familiarity and Predictability, (2) Cognitive Demands Before and During Shopping, (3) Sensory Overload and Time Pressure, and (4) Payment, Technology, and Feelings of Exposure.

#### Reliance on familiarity and predictability

Familiarity with place, layout, and routine seems to support independent retail engagement for PLWD. Specifically, familiarity with staff and the shopping environment supports feelings of safety, independence and confidence, reduced cognitive effort, and enabled individuals to anticipate task demands (Brorsson et al., 2011, 2013; Crampton and Eley, 2013; Silverman, 2021; Hebert, 2019). Conversely, changes such as product reorganisation, altered layouts, or revised packaging appear to disrupt established mental maps, rendering previously accessible environments unfamiliar and difficult to navigate (Brorsson et al., 2011, 2013, 2020; Quinn et al., 2022; Healthcare News, 2017). PLWD experienced difficulties navigating supermarkets when layouts were inconsistent or illogical (Brorsson et al., 2013; Quinn et al., 2022). Specifically, unexpected placement of products, multiple service points, or poorly organised checkout areas created confusion, increasing the likelihood of errors and stress (Brorsson et al., 2020). Further confusion arose when PLWD could not understand the organisation of a shop. In these circumstances, PLWD often searched for items in an unstructured way (e.g., walking in an unplanned, exploratory fashion with the hope of encountering the intended product) (Brorsson et al., 2013). Navigational difficulties en route to shops were also widely reported, including difficulties with left–right orientation, stress and anxiety, feelings of insecurity in public spaces, dizziness, mental fatigue (Brorsson et al., 2013). As a result, many participants restricted shopping to small, local shops and avoided larger supermarkets or unfamiliar retail spaces, contributing to reduced spatial mobility and choice (Brorsson et al., 2011, 2013; Wu et al., 2019; Juste, 2023).

#### Cognitive demands before and during shopping

The cognitive demands of retail engagement extend beyond the time within the store. Specifically, retail engagement was consistently described as beginning before leaving the home, with participants reporting difficulties remembering the purpose of the trip, gathering necessary items (e.g., keys, wallet, shopping list), and retaining task goals across transitions (Brorsson et al., 2011, 2013). Although compensatory strategies, such as written notes, were commonly used, reliance on these aids was experienced as precarious, as notes could be forgotten or misplaced (Brorsson et al., 2013). The quantity of available products to choose from placed additional cognitive demand, with PLWD reported feelings of overwhelm and confusion when there were many brands available to choose from Brorsson et al. (2013, 2020).

#### Sensory overload and time pressure

Sensory characteristics of retail environments were frequently reported as overwhelming, leading to reduced concentration and, in some cases, avoidance of shopping altogether (Brorsson et al., 2011, 2013; Silverman, 2021). Noise from music, announcements, and other shoppers reduced PLWDs ability to concentrate, while bright lighting and visually cluttered displays contributed to cognitive overload (Brorsson et al., 2020; Juste, 2023). Some PLWD also experienced visual illusions created by store interiors designed for aesthetic appeal (e.g., mirrors at vegetable stands giving the impression of more produce, or automatic glass doors and walls that were difficult to distinguish from the surrounding space) (Brorsson et al., 2020). Crowded shops and fast-paced environments, particularly during peak shopping hours, were also experienced as highly stressful (Brorsson et al., 2011, 2020). To manage these challenges, many participants adopted coping strategies such as shopping early in the morning to avoid crowds, limiting time spent in-store, or taking short breaks (Brorsson et al., 2011, 2020).

#### Payment, technology, and feelings of exposure

Payment systems and retail technologies were identified as significant barriers for PLWD. Self-service checkouts and card readers that required multiple steps and rapid responses caused stress and feelings of exclusion (Brorsson et al., 2011, 2013, 2020; Quinn et al., 2022; Juste, 2023). Difficulties with remembering PIN numbers, counting money, and managing queues often led to reliance on carers or avoidance of certain shops (Hebert, 2019; Quinn et al., 2022). Anticipation of these challenges frequently generated anxiety before entering the store, with some participants actively planning how to avoid self-service checkouts or selecting shops based on the availability of staffed tills (Brorsson et al., 2013).

### Support (interventions) that facilitate engagement with the retail industry for PLWD

#### Academic recommendations

Nine academic studies (Brorsson et al., 2013, 2020; Crampton and Eley, 2013; Silverman, 2021; Hebert, 2019; Wu et al., 2019; Ward et al., 2018; Igarashi et al., 2020; Quinn et al., 2022) detailed recommendations to support PLWD in retail settings. Recommendations can be broadly classified into staff training, environmental changes, and technology-related interventions.

With respect to staff training, supportive social interactions were consistently highlighted as essential, with PLWD prioritising shops where staff were patient, understanding, and willing to offer assistance when confusion arose (Crampton and Eley, 2013; Silverman, 2021; Hebert, 2019; Wu et al., 2019; Ward et al., 2018). Multiple studies emphasised the importance of dementia awareness training for retail staff to improve shopping experiences for PLWD. Such training should provide insight into what it is like to live with dementia, alongside developing an understanding of how environmental factors, such as spatial design or soundscapes, may exacerbate difficulties (Quinn et al., 2022). Training should also equip staff with skills to communicate effectively, respond appropriately in emergency situations, and provide support without stigma, thereby reducing stress and negative experiences during shopping (Wu et al., 2019; Igarashi et al., 2020; Quinn et al., 2022). Empathy and patience among staff were identified as particularly important in enabling PLWD to feel at ease within retail environments (Quinn et al., 2022).

Regarding environmental changes, studies recommended simplified and predictable store layouts, consistent product placement, and clearer signage to support navigation and reduce disorientation (Brorsson et al., 2013; Quinn et al., 2022). Suggested mitigation measures to further enhance concentration and reduce anxiety, included controlled lighting, reduced visual clutter, and the provision of quieter shopping periods (Brorsson et al., 2020; Silverman, 2021).

Finally, technology-related interventions primarily focused on reducing reliance upon complex technologies. Recommended strategies included the availability of staff-assisted checkouts, with staff trained to recognise when customers are struggling with financial tasks, alongside simplified payment processes and support for the use of cards or coins (Brorsson et al., 2013; Quinn et al., 2022). Brorsson et al. (2020) additionally proposed the introduction of “slow check-out” counters to reduce time pressure and stress by allowing more time and access to patient, supportive staff.

#### Practical guidance

Guidance on best practise for dementia-friendly retail consistently highlights the importance of both person-centred approaches through training, awareness, and empathetic interactions, and environmental adaptations, demonstrating that relatively small, practical changes can significantly improve accessibility and the shopping experience for people living with dementia.

Training emerged as a central recommendation with guides recommending that training should focus on practical communication techniques, including speaking clearly and slowly, using short sentences, and asking direct questions. Staff are encouraged to demonstrate empathy and respect, actively listen, maintain eye contact and maintain patience to create a welcoming and understanding environment for people living with dementia. Some guides explicitly advocated for formal dementia education and awareness training for all retail staff (The Carer, 2016; Bright Care, 2023; Southampton Data Observatory, n.d.; Dementia Friendly Iowa, 2024; Baker, 2022; Vermont Department of Health, n.d.; Forest of Dean Dementia Action Alliance, 2024; Green and Lakey, 2013; International Longevity Centre UK, 2024; Association of Convenience Stores, 2020; Dementia Australia, 2019). In comparison, other guides recommended role-based approaches, such as dementia champions with specific staff receiving advanced training and being identifiable through provision of a dementia champion badge (Southampton Data Observatory, n.d.; Vermont Department of Health, n.d.; Dementia Australia, 2019). Other guidance embedded training within broader principles of inclusive customer service and workforce wellbeing, framing training as a core component of age-friendly retail practise rather than a condition-specific intervention (The Carer, 2016; International Longevity Centre UK, 2024; Age UK, 2017; Association of Convenience Stores, 2020). Additionally, several guides provided detailed behavioural recommendations without explicitly referring to training, implicitly reinforcing the role of staff education in delivering dementia-friendly retail environments (Bould and Hill, 2018; Wootten et al., 2016; South Brent and District Caring, 2018; Wells, 2018; Wisconsin Department of Health Services, 2019; Alzheimer Scotland, 2025; Local Government Association, n.d.; Talk Dementia UK, n.d.).

Assisting customers was another central recommendation across guidance. Staff are encouraged to actively help customers move through the store and locate items, offering to accompany them along aisles rather than relying on verbal directions alone (South Brent and District Caring, 2018; The Carer, 2016; Dementia Friendly Iowa, 2024; Alzheimer Scotland, 2025; Local Government Association, n.d.; Talk Dementia UK, n.d.; Forest of Dean Dementia Action Alliance, 2024; Green and Lakey, 2013; Association of Convenience Stores, 2020; Dementia Australia, 2019). Guidance emphasises support at checkout, including helping with counting money, recognising coins and notes, completing card payments, simplifying choices, and providing designated staff or delivery options to reduce stress (South Brent and District Caring, 2018; The Carer, 2016; Wells, 2018; Dementia Friendly Iowa, 2024; Alzheimer Scotland, 2025; Local Government Association, n.d.; Talk Dementia UK, n.d.; Forest of Dean Dementia Action Alliance, 2024; Association of Convenience Stores, 2020). Guidance also highlights recognition of hidden disabilities, such as sunflower lanyards, to discreetly identify customers who may need extra assistance (Hidden Disabilities Sunflower, 2025). Furthermore, providing consistent support through familiar staff members is recommended (South Brent and District Caring, 2018; Dementia Friendly Iowa, 2024; Wisconsin Department of Health Services, 2019; Alzheimer Scotland, 2025).

Considering environmental adaptations, guidance included adjustments to store layout, signage, and wayfinding to improve accessibility (e.g., clear signs, colour coding, quiet spaces) (Bould and Hill, 2018; Wootten et al., 2016; The Carer, 2016; Southampton Data Observatory, n.d.; Wisconsin Department of Health Services, 2019; Forest of Dean Dementia Action Alliance, 2024; Green and Lakey, 2013; Association of Convenience Stores, 2020). Consistent product placement was highly recommended to support a sense of familiarity. Additional suggestions included acoustic improvements to reduce background noise, the provision of quite spaces, minimising visual clutter to reduce sensory overwhelm, as well as the provision of seating or rest areas and clearly marking accessible toilets (South Brent and District Caring, 2018; Bright Care, 2023; Southampton Data Observatory, n.d.; Alzheimer Scotland, 2025; Vermont Department of Health, n.d.; Age UK, 2017; Association of Convenience Stores, 2020; Dementia Australia, 2019).

#### Application of guidance in practise

Across the UK, and internationally, many retail companies have started to implement best practise guidelines to support customers living with dementia. Physical store adaptations, including the introduction of relaxed or slow shopping lanes for people with dementia or disabilities to provide more time to customers and encourage conversations with checkout workers, were frequently reported (Dawood, 2015; Team, 2018; North Central London Integrated Care System, n.d.; Abacare, 2018; Sale, 2015; Grant, 2017; Tate, 2018; Worcester News, 2017; Dementia Barnsley, 2018; Chandler, 2018; Press and Journal, 2017; Retail Customer Experience, 2020; The News, 2018). Additionally, shops have improved their layouts through changes to signage, by using contrasting colours and clear fonts for toilets, checkouts and store navigation, and embedding dementia friendly toilets within shops (Dawood, 2015; Co-op News, 2024; British Toilet Association, 2017; Avis-Riordan, 2017; Bowman, 2019). Specific retailers have also implemented initiatives, such as quiet or inclusive shopping hours in which noise level in shops is lowered and additional support is provided to customers (Motability Scheme, 2024; Munbodh, 2018; Robinson, 2019). Moreover, some retailers have specific dementia friendly shopping times in which dementia support organisations are present to offer advice and support on those days (Wood, 2024).

Staff education and training has also been widely implemented across the UK. For example, in 2021 the Association of Convenience shops in the UK estimated that around 4,500 independent shops had dementia friends working within their shops (Association of Convenience Stores, 2021). Major retailers have also embraced this approach participating in the Alzheimer’s society Dementia Friends training initiative, ensuring staff are equipped to support PLWD (Lawrence, 2018; Cassidy, 2021; Hawkins, 2019; Gilles, 2017; InYourArea Community, 2021; Co-op News, 2024; Green, 2023; LoveBelfast, 2018; Iceland Foods Charitable Foundation, n.d.; Marks & Spencer, n.d.; The Newsroom, 2018; The Newsroom, 2017; Retail Bulletin, 2014; The Newsroom, 2018; Hewitt, 2022; Lundberg, 2015).

### Methodological quality

Quality appraisal using the MMAT (Hong et al., 2018) indicated that, overall, included studies demonstrated appropriate data collection methods aligned with their research questions, accurate interpretation of findings, and coherence between data sources, collection, analysis, and interpretation. All studies met the MMAT criteria without methodological flaws. However, it is important to note that the MMAT serves as a screening tool focused on core methodological adequacy and does not capture all nuances of study quality or contextual limitations (see Supplementary Table S5 for the full methodological quality assessment). All included journals were ranked within Q1 or Q2 in Scimago journal and country ranking systems relevant to their respective disciplines, indicating that the studies were published in established and reputable peer-reviewed outlets (see Supplementary Table S3 for the full Scimago assessment).

## Discussion

This scoping review is the first to comprehensively synthesise evidence regarding the lived experiences of PLWD when engaging with the retail industry, alongside reviewing strategies designed to support these activities. In doing so, we identified key challenges faced by PLwD, including difficulties navigating shops, managing cognitive demands, coping with sensory overload, and interacting with complex payment systems, as well as the importance of familiarity, routine, and supportive social interactions. We also mapped a range of interventions and guidance, spanning staff training and environmental adaptations.

The included studies highlight how experiences of retail environments are shaped by a complex interplay of cognitive, sensory, and social factors. Difficulties navigating shops, managing cognitive demands, coping with sensory overload, and interacting with complex payment systems were consistently reported, reinforcing the idea that everyday retail environments, despite being a part of everyday community engagement, are not inherently designed to accommodate cognitive differences (Brorsson et al., 2011, 2013, 2020; Silverman, 2021; Wu et al., 2019; Juste, 2023). This highlights how barriers within retail settings can reduce the accessibility of community spaces for PLWD and thus undermine independence and community engagement. Familiarity and routine emerged as key facilitators of positive experiences, suggesting that predictability and prior knowledge of the environment reduce cognitive load and enable greater continued autonomy and participation within local communities (Brorsson et al., 2011, 2013; Crampton and Eley, 2013; Silverman, 2021; Hebert, 2019). Conversely, unpredictable layouts, inconsistent product placement, and complex technologies such as self-service checkouts increased stress, illustrating how seemingly minor environmental features can significantly impact autonomy for PLWD and contribute to withdrawal from community spaces (Brorsson et al., 2011, 2013, 2020; Juste, 2023; Healthcare News, 2017). Interestingly, these findings closely align with broader literature which similarly highlights the benefits of environmental predictability and reduced cognitive and sensory burden when designing environments that support autonomy for PLWD (Ghamari et al., 2025; Waller and Masterson, 2015; Siegelaar et al., 2025).

While these findings highlight important functional barriers and facilitators, focusing solely on these aspects risks overlooking the broader psychosocial significance of shopping for PLWD. Evidence from additional qualitative studies beyond the scope of this review (e.g., Clark and Phillipson, 2021; Fox et al., 2025a) suggests that shopping can serve as a therapeutic activity, providing distraction from the daily challenges of living with dementia and supporting continuity of identity. This broader perspective helps to contextualise why exploring barriers and facilitators is important and underpins the potential value of inclusive retail strategies, staff training, and environmental adaptations in enhancing the everyday experiences of PLWD.

Many initiatives currently in place, particularly those which involve environmental adaptations such as slow shopping lanes and quiet shopping hours, function as mechanisms that divide PLWD from the wider population. While these measures are intended to enhance accessibility, they may also inadvertently contribute to stigmatisation. Specifically, critics suggest that such interventions risk reinforcing a “them and us” divide, potentially undermining inclusive retail experiences for PLWD (Britton, 2016; Ianzito, 2025). Stigma is well established as a factor that can negatively influence outcomes for people living with dementia, including reduced engagement and reluctance to seek support or disclose needs. Specifically, within the Dementia Inequalities Model (Giebel, 2024), stigma operates as a key structural and social determinant shaping access to resources, service use, and help-seeking behaviour. From this perspective, retail adaptations that visibly single out PLWD may inadvertently exacerbate existing inequalities by discouraging participation or help-seeking, despite their intention to promote inclusion. Inclusive retail strategies may therefore benefit from moving beyond visible differentiation and instead prioritise universal, stigma-reducing approaches that support engagement and help-seeking among PLWD. Moreover, many dementia-friendly initiatives align with principles of universal design and may be better conceptualised as “human-friendly services” (Story, 2001). Environmental adaptations such as reduced noise and clearer signage may benefit a wide range of individuals, including older adults, people with sensory sensitivities, parents with young children, or those experiencing temporary stress, not solely PLWD. It is, however, important to note that these criticisms are not derived from PLWD and there is currently no empirical research exploring PLWD’s own perspectives on these initiatives, thus rendering this assumption purely speculative and positioned from the perspective of someone not living with dementia. Therefore, further research exploring PLWD’s perceptions of current initiatives is required to substantiate these assumptions before recommendations can be made.

Through the non-academic searches, we identified that, across the UK, many retail companies have begun implementing best practise guidelines to support PLWD. However, despite the widespread adoption of initiatives there remains a notable absence of formal evaluation. Specifically, to the best of the authors’ knowledge, to date, no studies have systematically measured the impact of these interventions on shopping independence or overall engagement for PLWD. Similarly, the long-term sustainability and effectiveness of environmental adaptations and staff training remain unknown. This gap limits our understanding of which interventions are most beneficial, how they interact, and how best to scale them in different retail contexts. Future research should, therefore, prioritise rigorous evaluation of existing dementia-friendly retail initiatives. Longitudinal quantitative or mixed-method studies should seek to assess the effectiveness of staff training and environmental modifications, in promoting autonomy, reducing cognitive and sensory burden, and improving overall shopping experiences for PLWD. Additionally, international comparisons are warranted to identify culturally and regionally relevant strategies, ensuring that interventions are both effective and contextually appropriate.

Critically, many of initiatives currently implemented in retail settings appeared to have been developed and introduced prior to empirical exploration of retail experiences for PLWD occurring. For example, the Alzhiemer’s Society in the UK issued dementia-friendly retail guidance in 2016 (Bould and Hill, 2018) which predates 70% of the academic articles included in this review (Silverman, 2021; Hebert, 2019; Wu et al., 2019; Ward et al., 2018; Igarashi et al., 2020; Quinn et al., 2022). Despite the lack of empirical evidence at the time, these practical interventions align closely with the recommendations emerging from the academic literature, including the importance of familiar environments, reduced sensory and cognitive load, and supportive social interactions. This congruence suggests that retail practise has been responsive to the lived experiences and practical needs of PLWD, even in the absence of a strong evidence base.

Despite the comprehensive search strategy, encompassing both academic and non-academic materials, and inclusion of many academic and non-academic resources [*n* = 74 (Academic *n* = 10; non-academic *n* = 64)], several limitations should be considered. First, non-academic materials were obtained through Google browser searches. As these platforms systematically prioritise search results based on your geographical location the non-academic materials included were predominantly UK-based guidance and records of implementation in practise in the UK, thus fundamentally limiting the generalisability of the present findings to alternative retail contexts in other countries and cultures. Consequently, this review should not be considered an exhaustive inventory of initiatives in place in retail settings globally, but rather a comprehensive synthesis of initiatives in place in the UK retail sector. Relatedly, these sources included guidance documents, organisational reports, and descriptions of implemented initiatives. While valuable for identifying emerging practise and implementation strategies, the evidentiary robustness of such materials varies. As such, findings derived from non-academic literature should be interpreted as indicative of practise developments rather than validated or generalisable evidence. Furthermore, all included academic materials employed qualitative methodologies with limited sample sizes. Therefore, caution should be exercised when generalising these findings to wider, potentially more diverse, populations.

## Conclusion

In summary, despite the centrality of retail environments to everyday life for PLWD, navigating shops, managing cognitive demands, coping with sensory overload, and interacting with complex payment systems are consistent barriers to independent and positive shopping experiences (i.e., Brorsson et al., 2011, 2013, 2020; Crampton and Eley, 2013; Silverman, 2021; Hebert, 2019; Wu et al., 2019; Ward et al., 2018; Igarashi et al., 2020; Quinn et al., 2022). Addressing these barriers requires both environmental and social interventions, including clear signage, predictable store layouts, reduced sensory and cognitive load, and well-trained, supportive staff. Despite widespread implementation of dementia-friendly initiatives, there remains a critical gap in rigorous evaluation of their effectiveness and long-term impact. Moreover, some initiatives risk inadvertently leading to stigmatisation of PLWD (Britton, 2016; Ianzito, 2025). Therefore, research must prioritise empirical assessments of these interventions, incorporating PLWD perspectives and international contexts to develop inclusive, sustainable retail practises. To support more effective retail engagement, national and sector-level policies should prioritise dementia-friendly practises, integrating evidence-based environmental adaptations and mandatory staff training, alongside monitoring and evaluation to ensure accessibility and inclusion are consistently achieved.

### Policy and practise implications

It is of note that the academic literature identified in this review originates from the fields of medicine, health and social science rather than business and marketing. This highlights a potential gap in cross disciplinary knowledge exchange and an area for future collaboration. Indeed, provisioning for an ageing population and, subsequently for PLWD, offers a market opportunity and a chance to for retailers to engage with corporate social responsibility (Cadburry, 2006). Current literature suggests that to truly serve PLWD, their needs must be conceptualised beyond the medical model, acknowledging the disabling effect of environments on social and retail access. Indeed, we should also recognise that many access barriers for PLWD are shared across consumer groups and are part of the ongoing discourse within the business and marketing literature, for example, choice overload and confusion in the shopping experience (Mitchell et al., 2023; Grandi and Cardinali, 2020). Thus, creating markets which support the needs of PLWD will likely improve the retail experience for a wider range of consumer groups, while potentially also avoiding stigmatisation from separating retail engagement for differing demographics.

In a recent scoping review of dementia experiences in the consumer marketplace, Primossi 2026 observed a similar gap in the literature, identifying an over-representation of health and social science articles in this field (Primossi et al., 2026). In this review Primossi suggests that health sciences often identify a need while simultaneously lacking the marketing expertise to develop viable solutions, while business literature simultaneously lacks sufficient clinical insights to create appropriate adaptations. Taking our findings alongside Primossi’s we suggest that it is important to view PLWD beyond the lens of their access barriers and instead as consumers with their own unique consumer identities and preferences. To successfully develop viable access solutions, we therefore need to combine the cross-disciplinary expertise of both business and marketing and health and social science professionals, supported by PLWD in a co-research capacity. Existing co-produced dementia friendly retail recommendations must be critically evaluated by marketing and business experts to develop commercially viable access solutions. Importantly, we need to work alongside PLWD and businesses to thoroughly evaluate these proposed solutions, holding space for iterations and changes where necessary. This will support retailers in engaging an expanding and increasingly important consumer demographic, while enabling PLWD to maintain agency, identity, and autonomy through everyday retail and consumer choices, alongside continued access to valued social and community third spaces.

## Footnotes

1
http://retractiondatabase.org/RetractionSearch.aspx
