# Supplementary material for: Dementia inclusive retail outlets: a scoping review of strategies to support people living with dementia in the retail industry
Source: Front Dement. 2026 Aug 17;5:1852323. doi: 10.3389/frdem.2026.1852323 (PMC13527713; doi:10.3389/frdem.2026.1852323)
Supplement: Supplementary file 1 [file Supplementary_file_1.docx]

**Supplementary Materials**

Contents

Table S1. Search Strings Applied to Academic Databases.	2

Table S2. Search Strings Applied for Non-academic Searching.	4

Table S3. Data Extraction for Academic Articles.	5

Table S4. Data Extraction for Non-academic Outputs.	9

Table S5. Assessment of Methodological Quality.	14

**Table S1. Search Strings Applied to Academic Databases.**

| Database | Search ID | Search String |
| --- | --- | --- |
| PubMed | S1  S2  S3  S4  S5 | "Dementia"[Title/Abstract] OR "Dementias"[Title/Abstract] OR "Alzheimer's disease"[Title/Abstract] OR "Alzheimer's"[Title/Abstract] OR Dement*[Title/Abstract] OR Alzheimer*[Title/Abstract] OR "AD"[Title/Abstract] OR "Rare Dementia"[Title/Abstract] OR "All-cause dementia"[Title/Abstract]  Shop*[Title/Abstract] OR "Shopping"[Title/Abstract] OR "Shops"[Title/Abstract] OR "Grocery Store"[Title/Abstract] OR "Groceries"[Title/Abstract] OR "Supermarket"[Title/Abstract] OR "Superstore"[Title/Abstract] OR "Market"[Title/Abstract]  "Friendly"[Title/Abstract] OR "Inclusive"[Title/Abstract] OR "Inclusion"[Title/Abstract] OR Community"[Title/Abstract] OR "Communities"[Title/Abstract] OR "Accessible"[Title/Abstract] OR Access*[Title/Abstract]  S2 OR S3  S1 AND S4 |
| PsycINFO | S1  S2  S3  S4  S5 | TI ( DE "Dementia" OR DE "Alzheimer's Disease" OR “Dementia” OR “Dementias” OR “Alzheimer’s disease” OR “Alzheimer’s” OR Dement* OR Alzheimer* OR “AD” OR “Rare Dementia” OR “All-cause dementia” ) OR AB ( DE "Dementia" OR DE "Alzheimer's Disease" OR “Dementia” OR “Dementias” OR “Alzheimer’s disease” OR “Alzheimer’s” OR Dement* OR Alzheimer* OR “AD” OR “Rare Dementia” OR “All-cause dementia” )  TI ( DE “Shop” OR DE “Retail” OR Shop* OR “Shopping” OR “Shops” or “Grocery Store” OR “Groceries” OR “Supermarket” OR “Superstore” OR “Market” ) OR AB ( DE “Shop” OR DE “Retail” OR Shop* OR “Shopping” OR “Shops” or “Grocery Store” OR “Groceries” OR “Supermarket” OR “Superstore” OR “Market” )  TI ( “Friendly” OR “Inclusive” OR “Inclusion” OR Community” OR “Communities” OR “Accessible” OR Access* ) OR AB ( “Friendly” OR “Inclusive” OR “Inclusion” OR Community” OR “Communities” OR “Accessible” OR Access* )  S2 OR S3  S1 AND S4 |
| CINHAL | S1  S2  S3  S4  S5 | TI ( (MH "Alzheimer's Disease") OR (MH "Dementia") OR “Dementia” OR “Dementias” OR “Alzheimer’s disease” OR “Alzheimer’s” OR Dement* OR Alzheimer* OR “AD” OR “Rare Dementia” OR “All-cause dementia” ) OR AB ( (MH "Alzheimer's Disease") OR (MH "Dementia") OR “Dementia” OR “Dementias” OR “Alzheimer’s disease” OR “Alzheimer’s” OR Dement* OR Alzheimer* OR “AD” OR “Rare Dementia” OR “All-cause dementia” )  TI ( (MH "Shopping") OR OR Shop* OR “Shopping” OR “Shops” or “Grocery Store” OR “Groceries” OR “Supermarket” OR “Superstore” OR “Market” ) OR AB ( (MH "Shopping") OR OR Shop* OR “Shopping” OR “Shops” or “Grocery Store” OR “Groceries” OR “Supermarket” OR “Superstore” OR “Market” )  TI ( “Friendly” OR “Inclusive” OR “Inclusion” OR Community” OR “Communities” OR “Accessible” OR Access* ) OR AB ( “Friendly” OR “Inclusive” OR “Inclusion” OR Community” OR “Communities” OR “Accessible” OR Access* )  S2 OR S3  S1 AND S4 |
| MEDLINE | S1  S2  S3  S4  S5 | XB ((MH "Dementia") OR (MH "Alzheimer Disease") OR “Dementia” OR “Dementias” OR “Alzheimer’s disease” OR “Alzheimer’s” OR Dement* OR Alzheimer* OR “AD” OR “Rare Dementia” OR “All-cause dementia”)  XB (Shop* OR “Shopping” OR “Shops” or “Grocery Store” OR “Groceries” OR “Supermarket” OR “Superstore” OR “Market”)  XB (“Friendly” OR “Inclusive” OR “Inclusion” OR Community” OR “Communities” OR “Accessible” OR Access*)  S2 OR S3  S1 AND S4 |
| EMBASE | S1  S2  S3  S4  S5 | “Dementia” OR “Dementias” OR “Alzheimer’s disease” OR “Alzheimer’s” OR Dement* OR Alzheimer* OR “AD” OR “Rare Dementia” OR “All-cause dementia”  OR Shop* OR “Shopping” OR “Shops” or “Grocery Store” OR “Groceries” OR “Supermarket” OR “Superstore” OR “Market”  “Friendly” OR “Inclusive” OR “Inclusion” OR Community” OR “Communities” OR “Accessible” OR Access*  S2 OR S3  S1 AND S4 |

**Table S2. Search Strings Applied for Non-academic Searching.**

Table S2. Grey literature search strings applied

| Search | Search String |
| --- | --- |
| S1 | Dementia friendly retail |
| S2 | Dementia friendly supermarkets |
| S3 | Dementia friendly shops |
| S4 | Barriers to supermarket shopping in dementia |
| S5 | Challenges to supermarket shopping in dementia |
| S6 | Barriers to retail in dementia |
| S7 | Challenges to retail in dementia |
| S8 | Shopping with dementia |
| S9 | Guidelines for dementia friendly shopping/retail |

**Table S3. Data Extraction for Academic Articles.**

| **Author** | **Journal published in (Scimago Journal & Country Rank)** | **Methods** | **Population (people living with dementia, carers) and Sample size (n)** | **Analytical focus** | **Key findings** |  |
| --- | --- | --- | --- | --- | --- | --- |
| Borsson et al. (2011) | Dementia (Q1 (Health (social science); Gerontology)) | Interviews | 7 PLWD | The experience of engaging with the retail industry for PLWD. | - PLWD relied heavily on familiar environments and routines to maintain independence whilst shopping. - Public shopping environments created significant barriers, including self-service technologies, crowded high-tempo settings, and noise, which increased stress. |  |
| Borsson et al. (2013) | Scandinavian Journal of Occupational Therapy (Q1 (Occupational Therapy)) | Interviews and participant observations. | 6 PLWD | The experience of engaging with the retail industry for PLWD.  Support (interventions) that facilitate engagement with the retail industry for PLWD. | - Participants reported frequent difficulties with memory and orientation. - Decision-making and payment were also challenging; too many product choices and restricted payment options (e.g., self-checkout, card PINs, counting cash) increased stress. |  |
| Brorsson et al. (2020) | Dementia (Q1 (Health (social science); Gerontology)) | Focus group interviews | 6 PLWD | The experience of engaging with the retail industry for PLWD.  Support (interventions) that facilitate engagement with the retail industry for PLWD. | - Experienced challenges when store layouts were illogical, payment technologies were complex, and products or services were arranged in unexpected ways. |  |
| Crampton et al. (2013) | Working with Older People (Q2 (Gerontology)) | Evaluation of dementia friendly community initiative. | Not reported | The experience of engaging with the retail industry for PLWD.  Support (interventions) that facilitate engagement with the retail industry for PLWD. | - People with dementia and their carers chose supermarkets even though this could mean a longer journey due to the guarantee of finding patient and sympathetic customer service, particularly at the checkout. |  |
| Silverman (2021) | Canadian Journal on Aging / La Revue canadienne du vieillissement (Q2 (Gerontology)) | Narrative methodological approach | 12 Carers | The experience of engaging with the retail industry for PLWD.  Support (interventions) that facilitate engagement with the retail industry for PLWD. | - Majority of carers explained that their choice of stores was based largely on social relationships and social connections. |  |
| Herbert (2019) | Dementia (Q1 (Health (social science); Gerontology)) | Interviews | 18 PLWD | The experience of engaging with the retail industry for PLWD.  Support (interventions) that facilitate engagement with the retail industry for PLWD. | - PWLD valued friendly and supportive staff whilst shopping. - People living with dementia often overlooked the extent of assistance provided by care partners, particularly in managing financial tasks. |  |
| Wu et al. (2019) | Journal of Advanced Nursing (Q1 (Nursing)) | Interviews | 16 PLWD 20 Carers  20 Caregivers’ care receivers | The experience of engaging with the retail industry for PLWD.  Support (interventions) that facilitate engagement with the retail industry for PLWD. | - Characteristics of dementia friendly stores include staff being knowledgeable on how to deal with PLWD especially in emergency cases. - Stressed importance of dementia education and training to staff. |  |
| Ward et al. (2018) | International Psychogeriatrics (Q1 (Geriatrics and Gerontology; Psychiatry and Mental Health)) | Longitudinal and comparative design | 21 individual (PLWD) or dyads (PLWD and carer) | Support (interventions) that facilitate engagement with the retail industry for PLWD. | - Participants highlight the benefits of retail spaces being dementia friendly. - Valued tables (in the café) that are really spread apart, open and light area. |  |
| Igarashi et al. (2020) | Journal of Applied Gerontology (Q1 (Gerontology)) | Interviews | 12 owners/managers of convenience stores who had been honoured for their excellence.  8 owners/managers or staff members | Support (interventions) that facilitate engagement with the retail industry for PLWD. | - Convenience store staff provided daily practical support to older customers, including help with shopping, carrying items, and managing payments. |  |
| Quinn et al. (2022) | Journal of Aging & Social Policy (Q2 (Gerontology; Public Administration)) | Open ended survey | 1,172 PLWD 702 Carers | The experience of engaging with the retail industry for PLWD.  Support (interventions) that facilitate engagement with the retail industry for PLWD. | - Expressed a desire for staff to receive training to enable them to be more ‘dementia-friendly.’ - Expressed that supermarket layouts are confusing. To reduce this issue clearer signage was suggested. |  |

**Table S4. Data Extraction for Non-academic Outputs.**

| **Author** | **Categorisation of paper** | **Key findings** |
| --- | --- | --- |
| Juste et al. (2023) | Report of difficulties faced | - Six key barriers for PWLD on the high street: mobility difficulties, limited choice, feeling misunderstood, anxiety in retail/leisure spaces, low confidence with finances, and challenges paying for goods. |
| Bould and Hill (2018) | Guide | - Practical guide for retailers to become more dementia-friendly by improving employee awareness and store environments. - Highlights simple, accessible changes that can significantly support people affected by dementia. |
| Wootten et al. (2016) | Guide | - A practical guide for retailers on supporting people with dementia through better staff understanding and improved store environments. - Explains that even small adjustments can greatly enhance accessibility and customer experience for those affected by dementia. |
| South Brent and district Caring (2018) | Guide | - Practical tips for retail staff: speak slowly, use short sentences, offer limited choices, and ask closed questions. - Use positive body language, reduce noise, assist with money or directions and maintain consistency in support while understanding memory limitations. |
| The Carer (2016) | Guide | - Highlights the importance of supporting people with dementia in shops and supermarkets, helping staff understand challenges like finding items, using facilities, handling money and navigating the store. |
| Bright Care (2023) | Guide | - Focuses on two areas: training staff to be patient, empathetic, clear, and responsive. - In the environment supermarkets should create supportive, accessible spaces. |
| Wells (2018) | Guide | - Practical measures like clear signage make shopping easier, while becoming dementia-friendly benefits businesses by retaining customers, attracting new ones and improving accessibility for all. |
| Southampton data observatory (n.d.) | Guide | - Importance of making spaces quieter, well-lit, clearly signed and easy to navigate. - Accessible seating, toilets and parking. - Staff-led changes include dementia training, proactive assistance with shopping and payments. |
| Dementia Friendly Iowa (2024) | Guide | - Implement dementia-friendly practices by preparing staff, learning local support and techniques. - Treat customers respectfully, involve care partners, assist with shopping and checkout and provide guidance if lost. |
| Wisconsin Department of Health Services (2019) | Guide | - Small changes make a big difference. - Clear signage, consistent layout. - Designate staff to accompany & support customers. - Stressed importance of staff awareness, understanding and patience. |
| Baker (2022) | Guide | - Improve accessibility by providing dementia training for staff, clear signage to support services, simplified checkout processes, and visual aids to help store navigation. |
| Alzheimer Scotland. (2025) | Guide | - Tips for retail staff to speak clearly, use friendly and respectful body language, listen carefully, be patient, reduce noise, ensure good lighting, assist with money and guide customers to items rather than giving directions. |
| Local Government Association (n.d.) | Guide | - Local authorities should consult people with dementia in building and public space design, using clear signage, colours, and layouts. - Staff guidance is to be patient, listen, assist with payments and navigation, and use pointing or writing to aid communication. |
| Vermont Department of Health (n.d.) | Guide | - Dementia-friendly retail supports employees who are caregivers with flexible schedules, sick leave and open communication. - Stores provide comfortable shopping for all, dementia training, clear signage, rest areas and non-slip flooring, universal symbols. |
| Talk Dementia UK (n.d.) | Guide | - Retailers should speak clearly, use short sentences, respond to emotions and assist with payments or holding items if needed. - Use eye contact, reduce choices, ask direct questions and display that staff are trained to support people with dementia. |
| The Forest of Dean Dementia Action Alliance (2024) | Guide | - Dementia-friendly checklist: staff trained, patient, use positive language; clear signage, fonts, and pricing; accessible routes, toilets, seating, and lifts; manageable noise and quiet shopping times; payment support available. |
| Green and Lakey (2013) | Guide | - People with dementia often give up activities, though shopping remains common. - Shops should raise awareness, train staff, and use feedback to improve accessibility and inclusivity. |
| Hidden Disabilities (2025) | Guide | - Detailed and accurate information is essential. - Surveyors visit every location, and data is informed by the user community. - Prompts use of sunflower lanyard for people with hidden disabilities. |
| International Longevity Centre UK (2024) | Guide | - Presents age- and dementia-friendly retail and hospitality guides, offering practical strategies like quiet shopping hours, staff training and accessibility adjustments to improve the experience for older customers across multiple businesses. |
| Age UK (2017) | Guide | - Age-friendly businesses should provide accessible toilets and seating, clear signage, well-designed websites, simplified and dementia-friendly checkouts, and reduce complex telephone menus. - Avoid assumptions about older shoppers, support navigation in-store and remove physical barriers. |
| Association of Convenience stores (2020) | Guide | - Recommends adapting store environments with good lighting, lower counters, simple payment processes, quiet spaces, fewer mirrors, clear signage, unobstructed layouts, accessible toilets, appropriate flooring, and reduced noise levels. |
| Dementia Australia (2019) | Guide | - Supermarkets should examine shop layout and have signage to ensure easy access. - Staff awareness training is very important. - Display dementia friendly symbol on shop front. - Includes an FAQ section with guidance on how to help PLWD in specific scenarios. |
| Association of Convenience Stores (2021) | Initiatives in stores | - Findings from ACS Voice of Local Shops Survey revealed that around 4,500 independent stores have Dementia Friends working in-store. - Webpage signposts to Dementia Friends training and "Supporting Vulnerable Customers" guide. |
| Lawrence (2018) | Initiatives in stores | - Retailers support dementia-friendly practice through staff training, Dementia Friends initiatives, assisted shopping, and pharmacy-based support. - Some also offer carer contact options, awareness campaigns, and commit to improving services for people living with dementia and their carers. |
| Cassidy (2021) | Initiatives in stores | - Havens Hospices in Essex has enrolled shop managers from their 23 stores onto the Dementia Friends course to help them and their staff recognise the signs of dementia and support their customers if they are vulnerable. - The course focuses on speaking quietly to PWLD and being patient when waiting for responses. |
| Hawkins (2019) | Initiatives in stores | - Braehead became Scotland’s first Dementia Friendly Shopping Centre through staff Dementia Friends training and partnership with Alzheimer Scotland, including use of the Purple Alert app to locate missing visitors. - Environmental changes include eye-level signage, coloured mats instead of black ones and traditional taps to improve familiarity and reduce confusion. |
| Dawood (2015) | Initiatives in stores | - Dementia friendly checkout launched by Tesco Chester Superstore - Checkout includes signage with clear, basic fonts, a simple message and the use of contrasting colours to enable the images and words to be distinct. |
| Gilles (2017) | Initiatives in stores | - In Bruges, shops are designated dementia friendly if employees are trained to recognise the condition and provide extra support to those customers. |
| Team (2018) | Initiatives in stores | - A local co-op branch had the family of a PLWD buy a gift card to be kept at the store for when he forgot to bring money. - Tesco's dementia friendly-checkouts with images of coins and values, and staff with dementia training. - Sainsburys' slow shopping scheme - also provided chairs so customers could sit. |
| North Central London Integrated Care System (n.d.) | Initiatives in stores | - The NCL Dementia Friendly scheme encourages supermarkets to complete awareness training and commit to practical changes. - Actions include improving signage and layout, offering slow shopping hours, supporting staff carers, and promoting inclusive community engagement. |
| InYourArea Community. (2021) | Initiatives in stores | - The initiative strengthens community integration and improves staff understanding of how to support customers living with dementia, as part of the wider Dementia Friendly Communities programme. |
| Abacare (2018) | Initiatives in stores | - Sainsbury’s introduced relaxed checkout lanes, greeters at store entrances, seating at aisle ends, and staff readily available to assist customers who may need extra support. |
| Co-op News. (2024) | Initiatives in stores | - Central England is the first to become official dementia friendly store. - Staff are given specialist training and also implemented special signing in the store. - The store aims to serve as a base to help train other staff. |
| Green (2023) | Initiatives in stores | - Edmonton Green Shopping Centre has trained its management team through Dementia Friends workshops, with staff registered as Dementia Friends. - They have shared information with retailers and developed a dementia-friendly map to support visitors. |
| LoveBelfast (2018) | Initiatives in stores | - The Junction Retail and Leisure Park became Northern Ireland’s first dementia-friendly shopping centre. - Staff completed dementia-friendly workshops, and each shop nominated a Dementia Friend to provide additional customer support. |
| Iceland (n.d.) | Initiatives in stores | - Iceland became the first food retailer to train all 30,000 colleagues as Dementia Friends. - It also introduced signage at tills encouraging customers to be patient and considerate towards people who may be living with dementia. |
| Maks & Spencer (n.d.) | Initiatives in stores | - The retailer committed to empowering all 80,000 colleagues to become Dementia Friends, embedding training and awareness across stores and offices through a national engagement campaign. |
| Sale (2015) | Initiatives in stores | - Tesco launched dementia friendly checkout following dementia friends training. - Includes pictures of coins and trained staff. - Signposted with large "Dementia Friendly Checkout" sign. |
| The News Room (2018) | Initiatives in stores | - The Junction Retail and Leisure Park set to become first dementia friendly shopping centre. - 85 staff offered dementia training. - Aim to train all new staff in future. |
| British Toilet Association (2017) | Initiatives in stores | - Sainsbury’s introduced dementia-friendly toilet signage across all stores, including large, colourful “way out” signs to reduce confusion and anxiety for customers with memory difficulties. |
| The News Room (2017) | Initiatives in stores | - Businesses are encouraged to interact positively with customers with dementia, and a recognisable logo is being introduced to identify supportive shops and spaces for carers to connect. |
| Grant (2017) | Initiatives in stores | - Tesco piloted a “relaxed” checkout in its Forres, Moray store, allowing customers to shop and pay at their own pace, reducing stress at the till. - Developed in partnership with Alzheimer Scotland, the initiative is open to all customers and aims to create a more supportive, pressure-free shopping experience. |
| Avis-Riordan (2017) | Initiatives in stores | - Sainsbury’s became the first supermarket to introduce dementia-friendly toilets, installing large, bright “way out” signs to improve navigation and reduce distress for customers with dementia. |
| Tate (2018) | Initiatives in stores | - Tesco Skipton introduced a “relaxed lane” checkout with large-print signs, extra space, and trained Dementia Champions to reduce stress for customers with dementia. - Supported by Alzheimer’s Society. |
| RetailBulletin (2014) | Initiatives in stores | - Argos, Homebase, Marks & Spencer, and Lloyds Banking Group committed to training over 121,500 staff as Dementia Friends to make UK high streets more dementia friendly. |
| Mobility Scheme (2024) | Initiatives in stores | - Discusses what UK supermarkets and shopping centres offer quiet hours with reduced music, dimmed lights, limited announcements, and calmer checkouts to create a more relaxed shopping experience for customers. |
| Worcester News (2017) | Initiatives in stores | - Tesco has introduced a “relaxed lane” at select stores, including Kidderminster, designed for slower, less stressful checkout experiences. The lane features wider space, decluttered aisles, dementia awareness-trained cashiers, information about dementia and a coin chart to help customers. |
| The News (2018) | Initiatives in stores | - Tesco North Harbour joined the Dementia Action Alliance to help make Portsmouth dementia-friendly. Staff are being trained to support customers with dementia, offering assistance with finding items, avoiding confusion, and paying for goods. |
| Hewitt (2022) | Initiatives in stores | - Stores train staff to recognise and support customers with dementia, helping them shop, pay and navigate calmly. - Training is delivered online and in-person, aiming to make public spaces easier and less stressful for people with dementia. |
| Dementia Barnsley (2018) | Initiatives in stores | - Tesco Supermarket in Penistone opened its first dementia friendly checkout with help from Barnsley Dementia Action Alliance and Making Space. |
| Bowman (2019) | Initiatives in stores | - Sainsburys implement dementia friendly toilets. - Result of a campaign by Angela Clayton-Turner, whose late husband had Alzheimer's |
| Chandler (2018) | Initiatives in stores | - Sainsbury’s trialling a ‘relaxed lane’ checkout with dementia-trained staff, hearing loop, and extra support. - Designed for shoppers who need more time, inspired by a previous ‘Slow Shopping’ trial. |
| Press and Journal (2017) | Initiatives in stores | - Tesco Scotland launched a relaxed checkout in Forres for customers needing extra time, including those with dementia, social anxiety, depression, or autism. - The lane allows shoppers to take their time and reduce stress at the checkout. |
| Munbodh (2018) | Initiatives in stores | - 8 major Asda stores trial an inclusive hour. - Offer calmer environment and additional support to customers for the first hour of the day. - Staff have undergone dementia and autism awareness training, and wear specific purple uniform. |
| Lundberg (2015) | Initiatives in stores | - Two supermarkets in The Hague train cashiers to recognise elderly customers needing help, with 20 staff trained by care providers. - Employees have contacts at care providers to help customers who forget their way home. |
| Robinson (2019) | Initiatives in stores | - ASDA runs a weekly “inclusive hour” with reduced noise for customers with dementia or sensory needs. - Staff receive dementia and autism awareness training, with charities providing support and feedback. |
| Retail Customer Experience (2020) | Initiatives in stores | - Tesco is piloting “relaxed” checkout lines for shoppers with dementia, Alzheimer’s or other illnesses. - Staff are trained to provide extra time and support, making the shopping experience less stressful. |
| Wood (2024) | Initiatives in stores | - Morrisons Little Waltham runs monthly dementia-friendly shopping sessions. - Visitors can enjoy tea and chat, with support and advice from dementia organizations like Alzheimer’s Society. |
| Healthcare News (2017) | Report of difficulties faced | - Details problems to overcome when food shopping with dementia. - Communication - support family member to ask for help whenever necessary. - Changing layouts, packaging, containers - recommend going to a familiar shop. - Recommend to always keep a shopping list. |

**Table S5. Assessment of Methodological Quality.**

| **Author** | **Is the qualitative approach appropriate to answer the research question?** | **Are the qualitative data collection methods adequate to address the research question?** | **Are the findings adequately derived from the data?** | **Is the interpretation of results sufficiently substantiated by data?** | **Is there coherence between qualitative data sources, collection, analysis and interpretation?** |
| --- | --- | --- | --- | --- | --- |
| Borsson et al. (2011) | Yes | Yes | Yes | Yes | Yes |
| Borsson et al. (2013) | Yes | Yes | Yes | Yes | Yes |
| Brorsson et al. (2020) | Yes | Yes | Yes | Yes | Yes |
| Crampton et al. (2013) | Yes | Yes | Yes | Yes | Yes |
| Silverman (2021) | Yes | Yes | Yes | Yes | Yes |
| Herbert (2019) | Yes | Yes | Yes | Yes | Yes |
| Wu et al. (2019) | Yes | Yes | Yes | Yes | Yes |
| Ward et al. (2018) | Yes | Yes | Yes | Yes | Yes |
| Igarashi et al. (2020) | Yes | Yes | Yes | Yes | Yes |
| Quinn et al. (2022) | Yes | Yes | Yes | Yes | Yes |
